# Exploring Pediatric Vertebral, Sacral, and Pelvic Osteosarcomas through the NCDB: Demographics, Treatment Utilization, and Survival Outcomes

**DOI:** 10.3390/children11081025

**Published:** 2024-08-21

**Authors:** Pemla Jagtiani, Mert Karabacak, Matthew T. Carr, Zeynep Bahadir, Peter F. Morgenstern, Konstantinos Margetis

**Affiliations:** 1 School of Medicine, State University of New York Downstate Health Sciences University, Brooklyn, NY 11203, USA; pemla.jagtiani@downstate.edu; 2 Department of Neurosurgery, Mount Sinai Health System, New York, NY 10029, USA; mert.karabacak@mountsinai.org (M.K.); matthew.carr@mountsinai.org (M.T.C.); peter.morgenstern@mountsinai.org (P.F.M.); 3 Department of Pediatrics, Weill Cornell Medicine, New York, NY 10065, USA; zeb4003@med.cornell.edu

**Keywords:** osteosarcoma, spine, pediatric, NCDB, disparity, survival

## Abstract

Background and Objectives: Retrieve data from the National Cancer Database (NCDB) to examine information on the epidemiological prevalence, treatment strategies, and survival outcomes of pediatric vertebral, sacral and pelvic osteosarcomas. Methods: We reviewed NCDB data from 2008 to 2018, concentrating on vertebral, sacral, and pelvic osteosarcomas in children 0 to 21 years. Our analysis involved logistic and Poisson regression, Kaplan-Meier survival estimates, and Cox proportional hazards models. Results: The study population included 207 patients. For vertebral osteosarcomas, 62.5% of patients were female, and 78.1% were white. Regional lymph node involvement predicted 80 times higher mortality hazard (*p* = 0.021). Distant metastasis predicted 25 times higher mortality hazard (*p* = 0.027). For sacral and pelvic osteosarcomas, 58.3% of patients were male, and 72% were white. Patients with residual tumor were 4 times more likely to have prolonged LOS (*p* = 0.031). No residual tumor (HR = 0.53, *p* = 0.03) and radiotherapy receipt (HR = 0.46, *p* = 0.034) were associated with lower mortality hazards. Distant metastasis predicted 3 times higher mortality hazard (*p* < 0.001). Hispanic ethnicity was linked to lower resection odds (OR = 0.342, *p* = 0.043), possibly due to language barriers affecting patient understanding and care decisions. Conclusions: In conclusion, our examination of NCDB offers a thorough exploration of demographics, treatment patterns, and results, highlighting the importance of personalized approaches to enhance patient outcomes.

## 1. Introduction

Osteosarcoma, characterized by malignant mesenchymal cells producing immature bone, is the most common primary malignant bone tumor [1,2]. Although osteosarcoma constitutes 30–80% of primary skeletal sarcomas, pediatric bone tumors are uncommon, with approximately 8.7 cases per million children per year, translating to about 650–700 cases annually in the United States (US) [3,4]. Osteosarcomas typically manifest in the long bones of the extremities, particularly in proximity to the metaphyseal growth plates [5]. The highest occurrence of primary osteosarcoma is seen during the second decade of life, indicating a correlation with the rapid proliferation of bone [6]. Spinal involvement is uncommon, representing only 3–5% of all osteosarcomas [7]. The infrequency and diverse associated factors of pediatric vertebral, sacral, and pelvic osteosarcomas present challenges in elucidating the presentation, optimal treatment, and probable outcomes for this disease in children.

Most studies of vertebral, sacral, and pelvic osteosarcomas are limited to small case series from single institutions [8,9]. This study seeks to fill this knowledge gap by utilizing the National Cancer Database (NCDB) to investigate the largest cohort of pediatric vertebral, sacral, and pelvic osteosarcomas to date. The primary objective is to determine prognostic factors by analyzing the epidemiology, treatment utilization, and survival outcomes of pediatric vertebral, sacral, and pelvic osteosarcomas.

## 2. Materials and Methods 

Patient demographics, tumor characteristics, treatments, and outcomes were all sourced from the NCDB. This study was exempted from institutional review board approval at the Icahn School of Medicine at Mount Sinai because it utilized deidentified patient data from the NCDB. The study adhered to the Strengthening the Reporting of Observational Studies in Epidemiology (STROBE) guidelines for reporting observational studies [10].

### 2.1. Study Population

The study cohort consisted of pediatric patients aged ≤21 years diagnosed with vertebral (mobile spine), sacral, and pelvic osteosarcomas between 2008 and 2018. Osteosarcomas were identified using the following International Classification of Diseases for Oncology, third edition (ICD-O-3) histology codes: 9180, 9181, 9182, 9183, 9184, 9185, 9186, 9187, 9192, 9193, 9194, and 9195. Vertebral localization was identified with ICD-O-3 topography code C41.2, and sacral and pelvic localization was identified using code C41.4. Individuals without survival data were excluded from the analysis.

### 2.2. Demographics and Tumor-Specific Variables

The following demographic and socioeconomic variables were analyzed: age, sex, race, Hispanic ethnicity, insurance status, percentage of non-high school graduates in zip code, median household income of zip code, residing population size, and Charlson-Deyo score as an indicator of existing comorbidities. Age was divided into three categories: 0–10 years, 11–15 years, 16–21 years. The 11–15 year category was chosen to represent the period typically associated with the adolescent growth spurt [11,12]. The 16–21 year category corresponds to a time when skeletal maturity is usually attained [13]. The 0–10 year age group was kept as a single category due to the limited number of cases within this range, which would make further subdivisions into smaller age groups statistically underpowered. Race was classified as white, black, or other, with the other category including any race that appeared fewer than 5 times in the study population. Insurance status was categorized as private, government-sponsored, or uninsured, indicating a patient’s primary coverage at diagnosis. The percentage of non-high school graduates in the zip code was categorized as ≤10.8% and >10.8%. Median household income within the zip code was split at $50,333. The population size of the area of residence was classified as either ≥250,000 (metro area) and <250,000 (urban/rural). A Charlson-Deyo score ≥ 1 signified the presence of at least one comorbidity. Tumor-specific variables analyzed included maximum tumor dimension, tumor grade (grades 1–2 versus grades 3–4), regional lymph node involvement, distant metastasis, and the American Joint Committee on Cancer (AJCC) stage (stages I, II, and III–IV). Maximum tumor dimension was categorized as ≤8 cm or >8 cm according to the NCDB database categories, although specific measurement details were not provided.

### 2.3. Treatment Data

Data on the following treatments were obtained: surgery, radiotherapy, and chemotherapy. Information on residual tumor post-resection was also collected and categorized by the presence or absence of residual tumor. Patients undergoing biopsy only without tumor resection were considered to not have undergone surgical resection. Radiotherapy modalities included external beam radiation, brachytherapy, radioisotope therapy, or any combination of these treatments. Chemotherapy was classified as either single agent or multi-agent regimens.

### 2.4. Outcomes of Interest and Statistical Analysis

The primary outcome assessed was overall survival (OS), defined as the time from initial diagnosis to death attributable to any cause. Secondary outcomes included: (1) the use of treatment modalities (surgery, radiotherapy, and chemotherapy) across all patients, and (2) short-term postoperative outcomes for the subset of patients who underwent sugery. OS rates for vertebral and sacral/pelvic osteosarcomas were visualized using Kaplan-Meier curves. Kaplan-Meier estimates of OS at 1, 5, and 10 years post-diagnosis were calculated separately for vertebral and sacral/pelvic osteosarcomas and compared using the log-rank test. The analyses were conducted separately for osteosarcomas in the vertebral region and sacral and pelvic regions.

Additional Kaplan-Meier curves were generated stratified by age category, stage, surgical resection status, radiotherapy status, and chemotherapy status. Log-rank tests compared differences in OS between these stratified groups. Univariate Cox proportional hazards regression models evaluated associations between patient and disease characteristics and mortality risk. To prevent collinearity, surgical resection status and residual tumor status were consolidated into a 3-level predictor (no surgery, surgery with no residual tumor, surgery with residual tumor). Cancer stage was not included as a variable; instead, maximum tumor dimension, tumor grade, regional lymph node involvement, and distant metastasis were incorporated separately, as these factors define stage determination. Covariates with *p* < 0.2 in univariate testing were included in a multivariate Cox proportional hazards model to determine independent predictors of mortality hazard.

Univariate and multivariate logistic regression models were utilized to analyze associations between patient/tumor characteristics and treatment utilization. These models focused on covariates that did not directly measure treatment or outcomes. Covariates with a *p*-value < 0.2 in univariate testing were included in the multivariate models to identify independent predictors of treatment utilization. Patients with incomplete data for a specific treatment were excluded from logistic regression analyses involving that treatment modality. For surgically-treated patients, the short-term postoperative outcomes analyzed included unplanned hospital readmissions within 30 days of discharge and prolonged hospital length of stay (LOS). For vertebral osteosarcomas, the prolonged LOS was defined as >7 days after definitive surgical resection, and for sacral and pelvic osteosarcomas, it was defined as >20 days after definitive surgical resection. Patients missing data on postoperative outcomes were excluded from related analyses.

For Cox proportional hazards models and logistic regression analyses, variables with more than 25% missing data were excluded from analysis. For variables with 25% or less missing data, multiple imputation by chained equations (MICE) was employed to fill in missing values before conducting the analyses [14]. A significance level (α) of 0.05 was preset for all hypothesis testing to indicate statistical significance. All data analyses were performed using R statistical software version 4.2.3 (2023; R Foundation for Statistical Computing, Vienna, Austria).

## 3. Results

**Figure 1:**
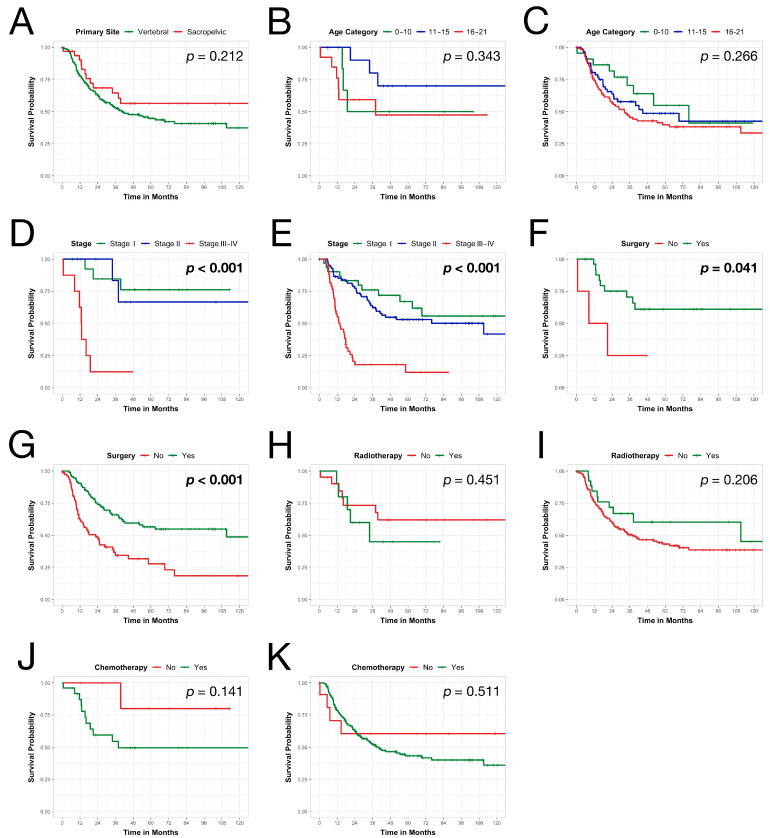
Kaplan-Meier survival estimates over time (**A**) grouped by primary site, (**B**) grouped by age category for vertebral osteosarcomas, (**C**) grouped by age category for sacral and pelvic osteosarcomas, (**D**) grouped by stage for vertebral osteosarcomas, (**E**) grouped by stage for sacral and pelvic osteosarcomas, (**F**) grouped by surgery receipt for vertebral osteosarcomas, (**G**) grouped by surgery receipt for sacral and pelvic osteosarcomas, (**H**) grouped by radiotherapy receipt for vertebral osteosarcomas, (**I**) grouped by radiotherapy receipt for sacral and pelvic osteosarcomas, (**J**) grouped by chemotherapy receipt for vertebral osteosarcomas, (**K**) grouped by chemotherapy receipt for sacral and pelvic osteosarcomas.

The study population included 207 pediatric patients diagnosed with vertebral (*n* = 32, 15.5%) and sacral/pelvic (*n* = 175, 84.5%) osteosarcomas using data collected from the NCDB between 2008–2018 (Table 1). The Kaplan-Meier estimated OS rates for vertebral osteosarcomas were 90.1% [95% confidence interval (CI) 80–97.2%] at 1 year, 94.4% (95% CI 48.8–85%) at 3 years, and 56.4% (95% CI 40.4–78.7%) at 5 and 10 years (Figure 1A). For sacral and pelvic osteosarcomas, the rates were 77.7% (95% CI 71.7–84.2%) at 1 year, 52.6% (95% CI 45.3–61%) at 3 years, 44.7% (95% CI 37.2–53.7%) at 5 years, and 37.3% (95% CI 28.4–49%) at 10 years. The difference in OS between vertebral and sacral/pelvic sites was not statistically significant per log-rank test (*p* = 0.212, Figure 1A).

### 3.1. Vertebral Osteosarcomas

This cohort comprised 32 patients with vertebral osteosarcomas (Table 1). The majority of individuals belonged to the older age groups, with 40.6% (*n* = 13) ages 11–15 years and 40.6% (*n* = 13) ages 16–21 years. Age category did not show a statistically significant difference in OS (*p* = 0.343, Figure 1B). More than half were female (*n* = 20, 62.5%). Most patients were white (*n* = 25, 78.1%) and 90.6% (*n* = 29) were not of Hispanic ethnicity. Of the patients, 33.3% (*n* = 10) had government-sponsored insurance while 66.7% (*n* = 20) had private insurance. Most patients resided in zip codes with ≤10.8% non-high school graduates (*n* = 15, 57.7%). Meanwhile, 38.5% (*n* = 10) were in the higher median income category (>$50,333) and most lived in metro areas with ≥250,000 residents (*n* = 20, 66.7%). Only one patient (3.1%) had comorbid conditions (Charlson-Deyo score ≥ 1). Most tumors were ≤8 cm (*n* = 20, 64.5%) and classified as grade 3–4 (*n* = 18, 56.2%), with no nodal involvement (*n* = 29, 90.6%), no distant metastasis (*n* = 27, 84.4%), and categorized as stage I (*n* = 14, 43.8%). OS differed significantly between stage I, II, and III–IV (*p* < 0.001, Figure 1D). The 5-year OS was 76.2% for stage I, 66.7% for stage II, and 12.5% for stage III–IV.

**Figure 2:**
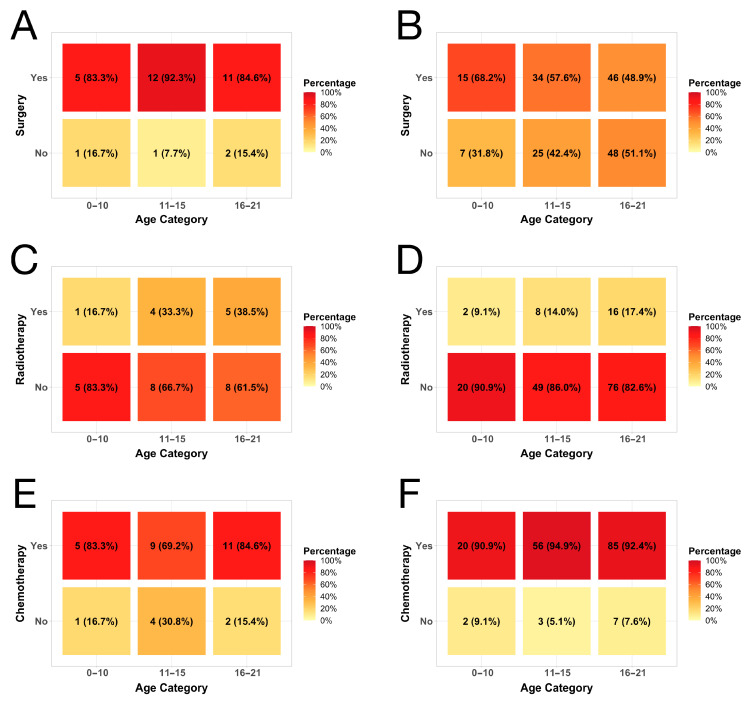
Heatmaps depicting the rates of (**A**) surgery by age category for vertebral osteosarcomas, (**B**) surgery by age category for sacral and pelvic osteosarcomas, (**C**) radiotherapy by age category for vertebral osteosarcomas, (**D**) radiotherapy by age category for sacral and pelvic osteosarcomas, (**E**) chemotherapy by age category for vertebra osteosarcomas, (**F**) chemotherapy by age category for sacral and pelvic osteosarcomas.

Approximately 87.5% (*n* = 28) of patients underwent surgical resection (Figure 2A). Among those resected, most achieved resection with no residual tumor (*n* = 14, 53.8%). OS differed significantly between resected and non-resected groups (*p* = 0.041, Figure 1F). Only 32.3% (*n* = 10) received radiotherapy (Figure 2C). Radiotherapy showed no significant association with OS compared to non-radiotherapy groups (*p* = 0.451, Figure 1H). Meanwhile, 78.1% (*n* = 25) received chemotherapy (Figure 2E). Similarly, chemotherapy demonstrated no significant difference in OS versus non-chemotherapy groups (*p* = 0.141, Figure 1J). Multivariate analyses found no statistically significant patient or tumor factors associated with utilization of these treatment modalities (Table 2). 

**Figure 3:**
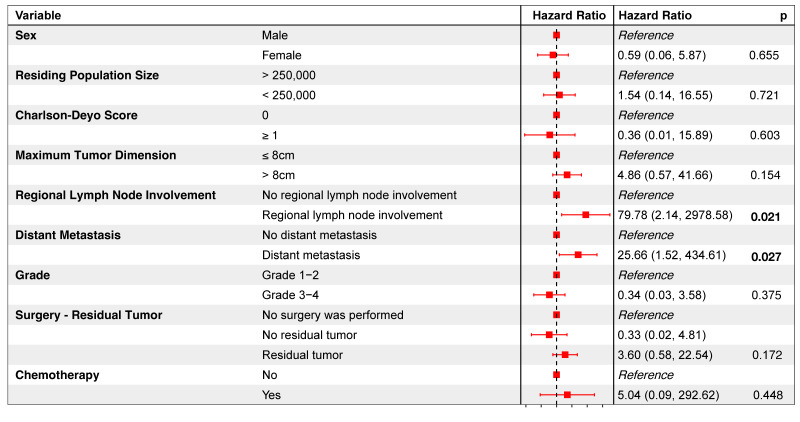
Multivariate Cox proportional hazards model evaluating the impact of selected variables (with *p*-values < 0.2 from a preliminary univariate Cox proportional hazards analysis) on the mortality risk of patients with vertebral osteosarcomas.

Among the 28 patients who underwent surgical resection, only 4 (14.3%) experienced unplanned readmissions (Table 1). Multivariate analysis did not identify any variables significantly associated with odds of unplanned readmissions or prolonged LOS (Supplementary Tables S1 and S2). When covariates with a *p* < 0.2 in univariate testing (Supplementary Figure S1) were included in a multivariate Cox proportional hazards model, regional lymph node involvement and distant metastasis were found to be significantly associated with a higher mortality risk (Figure 3). Regional nodal involvement predicted nearly 80 times higher mortality hazard [Hazard Ratio (HR) = 79.78, *p* = 0.021)]. Meanwhile, distant metastasis predicted over 25 times higher mortality hazard (HR = 25.66, *p* = 0.027).

### 3.2. Sacral and Pelvic Osteosarcomas

This cohort comprised 175 patients with sacral and pelvic osteosarcomas. The majority were in the oldest age group, with 53.7% (*n* = 94) ages 16–21 years. Age category did not associate with overall survival (OS) difference (*p* = 0.266, Figure 1C). Over half were male (*n* = 102, 58.3%). Most were white (*n* = 126, 72%) and non-Hispanic (*n* = 144, 85.2%). Only 3% (*n* = 5) lacked insurance, while private insurance covered 58.3% (*n* = 98). Most resided in zip codes with >10.8% non-high school graduates (*n* = 85, 52.8%). Meanwhile, 42.9% (*n* = 69) were in higher median income areas (>$50,333), and most lived in metro regions (*n* = 138, 85.2%). Eighteen patients (10.3%) had comorbidities (Charlson-Deyo score ≥ 1). Majority of tumors were >8 cm (*n* = 106, 69.3%) and high grade (*n* = 127, 80.4%), with no regional lymph node involvement (*n* = 147, 95.5%) and no distant metastases (*n* = 124, 77.5%). Over half were stage II (*n* = 82, 52.6%). OS differed significantly between stages (*p* < 0.001, Figure 1E), with 5-year OS of 67% for stage I, 53% for stage II, and 12% for stage III–IV.

Over half of patients (*n* = 95, 54.3%) underwent surgical resection (Figure 2B). Among those resected, most achieved no residual tumor (*n* = 60, 75%). Overall survival (OS) differed significantly between resected and non-resected groups (*p* < 0.001, Figure 1G). Only 15.2% (*n* = 26) received radiotherapy (Figure 2D). Radiotherapy did not demonstrate a significant difference in OS compared to non-radiotherapy (*p* = 0.206, Figure 1I). Meanwhile, most received chemotherapy (*n* = 161, 93.1%, Figure 2F), which did not associate with OS difference (*p* = 0.511, Figure 1K). Multivariate logistic regression analysis showed Hispanic ethnicity (OR = 0.342; *p* = 0.043) and distant metastases (OR = 0.173; *p* < 0.001) were less likely to undergo resection. No variables significantly predicted radiotherapy odds (Supplementary Table S3). Finally, tumors > 8 cm had 4 times higher chemotherapy odds (OR = 4.037; *p* = 0.026, Supplementary Table S4). 

**Figure 4:**
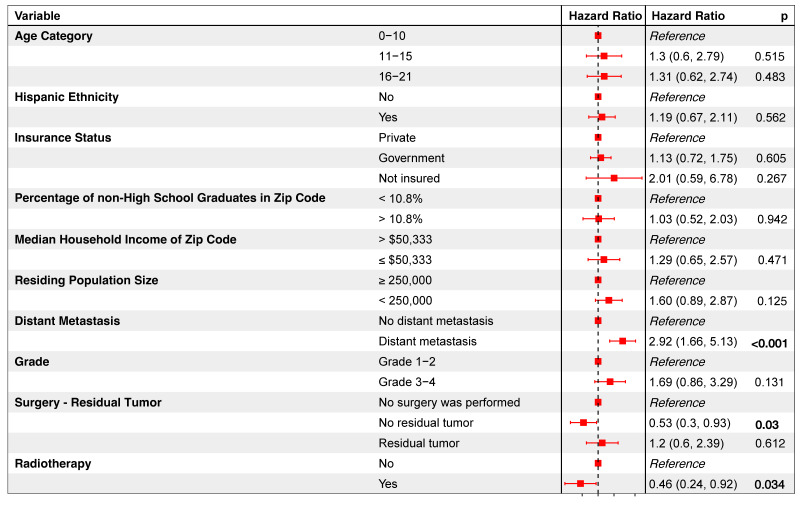
Multivariate Cox proportional hazards model evaluating the impact of selected variables (with *p*-values < 0.2 from a preliminary univariate Cox proportional hazards analysis) on the mortality risk of patients with sacral and pelvic osteosarcomas.

Of 95 resected patients, only 4 (2.3%) had unplanned readmissions (Table 1). Both univariate and multivariate analyses identified no variables significantly associated with the odds of readmission (Supplementary Table S1). Meanwhile, multivariate analysis showed residual tumor status increased prolonged length of stay odds over 4-fold (OR = 4.389; *p* = 0.031, Supplementary Table S2). After including univariate covariates with *p* < 0.2 (Supplementary Figure S2) into a multivariate Cox model, distant metastasis predicted nearly 3 times the mortality hazard (HR = 2.92, *p* < 0.001, Figure 4). Conversely, no residual tumor (HR = 0.53, *p* = 0.03) and radiotherapy receipt (HR = 0.46, *p* = 0.034) were associated with lower mortality hazards.

## 4. Discussion

Vertebral, sacral and pelvic osteosarcomas present distinct challenges in pediatric cases. However, research on clinical patterns and outcomes for this population remains limited. Therefore, this study aimed to address gaps in knowledge by utilizing NCDB data to elucidate the epidemiology, treatments, and survival rates pertaining specifically to pediatric vertebral and sacral/pelvic osteosarcomas from 2008–2018. While the NCDB has been used to investigate primary tumors of the spine broadly [15,16,17], there has been no focus on isolating the pediatric subgroup with osteosarcomas. Centering the analysis on pediatrics facilitates critical insights, given the considerable differences in disease manifestation and prognosis between children and adults. By exclusively examining this population, the current study provides fundamental epidemiologic and outcome data to improve the overall understanding of pediatric vertebral and sacral/pelvic osteosarcomas.

### 4.1. Demographic and Socioeconomic Factors 

Hispanic ethnicity was associated with a decreased likelihood of undergoing resection (OR = 0.342, *p* = 0.043) for sacral and pelvic osteosarcomas (Table 2). This aligns with Goulding et al. findings from analyzing 1415 osteosarcoma patients in the Surveillance, Epidemiology, and End Results (SEER) database [18]. Their research found that Hispanic ethnicity notably correlated with higher metastatic rates at diagnosis. Moreover, they highlighted limited English proficiency as a significant barrier contributing to delayed diagnosis and inferior outcomes from reduced access to appropriate medical care [18]. Such language barriers potentially explaining patient hesitancy in receiving care or fully understanding their diagnosis may account for the lower resection odds seen among patients with Hispanic ethnicity in this cohort.

### 4.2. Tumor Characteristics 

We observed significant associations between the stage of osteosarcomas and OS. OS differed significantly between stages I, II and III–IV in both tumor sites. Moreover, regional lymph node involvement and distant metastases emerged as mortality risk indicators. For vertebral osteosarcomas, regional lymph node involvement was associated with a nearly 80-fold increase in mortality risk, while distant metastasis was linked to a nearly 26-fold increase. Similarly, distant metastasis correlated with almost a 3-fold rise in mortality risk for sacral and pelvic osteosarcomas. These align with literature consistently showing survival differences by stage, nodes, and metastases [9,19,20]. Spinal osteosarcoma appears more likely to metastasize at the time of diagnosis compared to limb tumors, potentially reflecting delayed diagnosis or age-specific differences in tumor biology [21]. Furthermore, early spinal osteosarcoma detection proves challenging as localized pain often constitutes the initial or only symptom, frequently mistaken as benign, causing delayed diagnosis and heightened metastatic risk [22]. This diagnostic challenge is further complicated by the presence of other differential diagnoses that can present with symptoms similar to those of osteosarcomas, such as symptomatic synovial cysts. Scrofani et al. discusses the spontaneous resolution of symptomatic synovial cysts of the lumbar spine and emphasizes that other conditions, such as herniated discs, spinal stenosis, or tumors, can present with similar symptoms [23]. Accurately distinguishing these pathologies is crucial, especially in pediatric cases, to prevent unnecessary interventions and the significant consequences that can arise from misdiagnosis. 

### 4.3. Treatment 

Surgical resection remains pivotal for the management of vertebral and sacral/pelvic osteosarcomas. Typically, these manifest as poorly defined vertebral lesions with lytic and sclerotic components [17]. The main goal of surgery is to achieve complete tumor removal with wide margins. In this cohort, 87.5% of vertebral and 54.3% of sacral and pelvic cases underwent resection. Sacral and pelvic osteosarcoma patients with distant metastases had lower resection odds. Despite most resections achieving no residual tumor, those with residuals had over 4 times the odds of prolonged hospitalization for sacral and pelvic tumors. Due to the locally aggressive nature of osteosarcoma, inadequate excision caries a high risk of recurrence and potential metastasis. Therefore, a wide resection is widely considered the optimal treatment option [24].

The intricate nature of vertebral and sacral/pelvic osteosarcomas, together with their rare occurrence and proximity to vital structures, poses significant treatment challenges [4]. Given these complexities, radiation therapy as the primary local treatment is typically reserved for surgically infeasible cases, proving beneficial for local disease control [14,15]. In this cohort, the low radiotherapy prevalence, seen in only 32.3% of vertebral and 15.2% of sacral/pelvic cases, highlights its cautious, selective application. Despite radiotherapy’s significant role in certain cases, our analysis suggested lowered mortality hazard association only for sacral and pelvic tumors. Another compelling finding was the link between comorbidities and decreased radiotherapy likelihood for sacral and pelvic osteosarcomas. This suggests that while radiotherapy may provide valuable local disease control, its influence on broader survival could be nuanced and context-dependent, warranting prospective confirmation.

The majority of patients received chemotherapy for both vertebral and sacral/pelvic osteosarcomas. In our study, patients with tumor dimensions >8 cm were 4 times more likely to undergo chemotherapy. Despite the significant role of chemotherapy in certain cases, our analysis suggested that its administration did not significantly impact OS for both vertebral and sacral/pelvic osteosarcomas. These findings collectively highlight the intricate decision-making process in treating vertebral and sacral/pelvic osteosarcomas.

### 4.4. Limitations

While this study offers novel insights into pediatric vertebral and sacral/pelvic osteosarcomas, it is essential to acknowledge certain inherent limitations associated with the use of NCDB. Notably, the NCDB reports mortality of all causes rather than reporting the specific cause of mortality and lacks detailed information on radiation/chemotherapy dosing, volumes, or treatment planning. Additionally, the database is limited to hospitals accredited by the Commission on Cancer, which may introduce selection bias. Despite these constraints, the NCDB remains a valuable resource for comprehending clinical characteristics, treatment modalities, and survival outcomes for rare cancers like pediatric vertebral and sacral/pelvic osteosarcomas, where conducting randomized controlled trials may be impractical. The database provides a foundation for exploring trends and patterns in patient demographics and treatment responses. To enhance the robustness of these findings, future investigations leveraging alternative population-based registries are warranted. Such studies may contribute to corroborating and expanding upon the insights gained from the NCDB, offering a more comprehensive understanding of the complexities surrounding pediatric osteosarcomas and their management.

## 5. Conclusions

This study leveraging NCDB data provides insights into pediatric vertebral and sacral/pelvic osteosarcomas across demographics, care patterns, and outcomes. We identified tumor lymph node involvement and distant metastases as pivotal prognostic mortality risk indicators. Moreover, substantial overall survival differences emerged between surgically resected and non-resected groups. Integrating demographics, tumor characteristics, and surgical intervention’s critical impact contributes a nuanced understanding to guide future advancements. Beyond treatment considerations, differences in resection rates among Hispanic patients highlight an opportunity to improve equitable access by addressing barriers. More broadly, this analysis provides a robust foundation for developing enhanced prognostic models and therapeutic approaches tailored to pediatric patients with these rare spinal tumors. Ultimately, these multifaceted insights hold promise to inform clinical decision-making, policies, and research directions–coalescing to enhance care standards and outcomes for children with vertebral or sacral/pelvic osteosarcomas.

## Figures and Tables

**Table 1:** Patient characteristics (IQR, interquartile range; S, surgery; CT, chemotherapy; RT, radiotherapy).

Variable		Vertebral (*n* = 32)		Sacropelvic (*n* = 175)	
		*n* (%) or Median (IQR)	Missing Data (%)	*n* (%) or Median (IQR)	Missing Data (%)
Year of Diagnosis	2008	2 (6.2%)	-	16 (9.1%)	0 (0%)
	2009	2 (6.2%)		9 (5.1%)	
	2010	2 (6.2%)		17 (9.7%)	
	2011	4 (12.5%)		23 (13.1%)	
	2012	2 (6.2%)		10 (5.7%)	
	2013	5 (15.6%)		13 (7.4%)	
	2014	-		9 (5.1%)	
	2015	5 (15.6%)		22 (12.6%)	
	2016	4 (12.5%)		22 (12.6%)	
	2017	4 (12.5%)		15 (8.6%)	
	2018	2 (6.2%)		19 (10.9%)	
Age		15 (12.75–19)	-	17 (14–19)	-
Age Category	0–10	6 (18.8%)	-	22 (12.6%)	0 (0%)
	11–15	13 (40.6%)		59 (33.7%)	
	16–21	13 (40.6%)		94 (53.7%)	
Sex	Male	12 (37.5%)	-	102 (58.3%)	0 (0%)
	Female	20 (62.5%)		73 (41.7%)	
Race	White	25 (78.1%)	-	126 (72%)	0 (0%)
	Black	4 (12.5%)		37 (21.1%)	
	Other	3 (9.4%)		12 (6.9%)	
Hispanic Ethnicity	No	29 (90.6%)	-	144 (85.2%)	6 (3.4%)
	Yes	3 (9.4%)		25 (14.8%)	
Insurance Status	Private insurance	20 (66.7%)	2 (6.2%)	98 (58.3%)	7 (4%)
	Government	10 (33.3%)		65 (38.7%)	
	Not insured	-		5 (3%)	
Percentage of non-High School Graduates by Zip Code	≤10.8%	15 (57.7%)	6 (18.8%)	76 (47.2%)	14 (8%)
	>10.8%	11 (42.3%)		85 (52.8%)	
Median Income by Zip Code	>$50,333	10 (38.5%)	6 (18.8%)	69 (42.9%)	14 (8%)
	≤$50,333	16 (61.5%)		92 (57.1%)	
Residing Population Size	≥250,000	20 (66.7%)	2 (6.2%)	138 (85.2%)	13 (7.4%)
	<250,000	10 (33.3%)		24 (14.8%)	
Charlson-Deyo Score	0	31 (96.9%)	-	157 (89.7%)	0 (0%)
	≥1	1 (3.1%)		18 (10.3%)	
Maximum Tumor Dimension	≤8 cm	20 (64.5%)	1 (3.1%)	47 (30.7%)	22 (12.6%)
	>8 cm	11 (35.5%)		106 (69.3%)	
Grade	Grade 1–2	14 (43.8%)	-	31 (19.6%)	17 (9.7%)
	Grade 3–4	18 (56.2%)		127 (80.4%)	
Regional Lymph Node Involvement	No	29 (90.6%)	-	147 (95.5%)	21 (12%)
	Yes	3 (9.4%)		7 (4.5%)	
Distant Metastasis	No	27 (84.4%)	-	124 (77.5%)	15 (8.6%)
	Yes	5 (15.6%)		36 (22.5%)	
Stage	Stage I	14 (43.8%)	-	31 (19.9%)	19 (10.9%)
	Stage II	10 (31.2%)		82 (52.6%)	
	Stage III–IV	8 (25%)		43 (27.6%)	
Surgery	No	4 (12.5%)	-	80 (45.7%)	0 (0%)
	Yes	28 (87.5%)		95 (54.3%)	
Residual Tumor *	No residual tumor	14 (53.8%)	2 (7.1%)	60 (75%)	15 (15.8%)
	Residual tumor	12 (46.2%)		20 (25%)	
Radiotherapy	No	21 (67.7%)	1 (3.1%)	145 (84.8%)	4 (2.3%)
	Yes	10 (32.3%)		26 (15.2%)	
Chemotherapy	No	7 (21.9%)	-	12 (6.9%)	2 (1.1%)
	Yes	25 (78.1%)		161 (93.1%)	
Treatment Combinations	S + CT + RT	8 (25.8%)	1 (3.1%)	7 (4.3%)	14 (8%)
	S + CT	12 (38.7%)		82 (50.9%)	
	S + RT	1 (3.2%)		-	
	CT + RT	1 (3.2%)		19 (11.8%)	
	S only	6 (19.4%)		4 (2.5%)	
	CT only	3 (9.7%)		49 (30.4%)	
	RT only	-		-	
Length of Stay *		5 (3–7.5)	1 (3.6%)	11 (6.8–20)	19 (20%)
Unplanned Readmission *	No	24 (85.7%)	-	87 (95.6%)	4 (4.2%)
	Yes	4 (14.3%)		4 (4.4%)	
1-Year Survival	No	3 (10.3%)	3 (9.4%)	38 (22.9%)	9 (5.1%)
	Yes	26 (89.7%)		128 (77.1%)	
3-Year Survival	No	10 (38.5%)	6 (18.8%)	77 (52.7%)	29 (16.6%)
	Yes	16 (61.5%)		69 (47.3%)	
5-Year Survival	No	12 (63.2%)	13 (40.6%)	86 (67.7%)	48 (27.4%)
	Yes	7 (36.8%)		41 (32.3%)	
10-Year Survival	No	12 (92.3%)	19 (59.4%)	90 (91.8%)	77 (44%)
	Yes	1 (7.7%)		8 (8.2%)	

* The reported data pertains only to patients who underwent surgical resection.

**Table 2:** Factors associated with the likelihood of surgical resection (CI, confidence interval; NA: not available; Inf, infinity).

	Variable		Vertebral		Sacropelvic	
			Odds Ratio (95% CI)	*p* Value	Odds Ratio (95% CI)	*p* Value
Univariate Logistic Regression	Age	0–10	Reference		Reference	
		11–15	2.4 (0.084–69.428)	0.562	0.635 (0.215–1.744)	0.389
		16–21	1.1 (0.045–14.418)	0.943	0.447 (0.158–1.162)	0.109
	Sex	Male	Reference		Reference	
		Female	0.515 (0.024–4.63)	0.586	0.645 (0.351–1.179)	0.155
	Race	White	Reference		Reference	
		Black	0.409 (0.035–9.73)	0.495	0.844 (0.404–1.768)	0.652
		Other	NA (0–Inf)	0.996	0.800 (0.238–2.687)	0.712
	Hispanic Ethnicity	No	Reference		Reference	
		Yes	0.037 (0.001–0.536)	0.021	0.317 (0.123–0.752)	0.012
	Insurance Status	Private insurance	Reference		Reference	
		Government	NA (0–Inf)	0.996	0.585 (0.314–1.081)	0.088
		Not insured	-		2.780 (0.394–55.436)	0.368
	Percentage of non-High School Graduates by Zip Code	≤10.8%	Reference		Reference	
		>10.8%	0.647 (0.069–6.045)	0.685	0.436 (0.235–0.798)	0.008
	Median Household Income by Zip Code	>$50,333	Reference		Reference	
		≤$50,333	1.941 (0.216–42.063)	0.586	0.583 (0.317–1.066)	0.081
	Residing Population Size	≥250,000	Reference		Reference	
		<250,000	NA (0–Inf)	0.996	0.682 (0.291–1.574)	0.369
	Charlson-Deyo Score	0	Reference		Reference	
		≥1	NA (0–Inf)	0.995	0.826 (0.307–2.222)	0.7
	Maximum Tumor Dimension	≤8 cm	Reference		Reference	
		>8 cm	0.158 (0.007–1.426)	0.131	0.613 (0.310–1.187)	0.151
	Grade	Grades 1–2	Reference		Reference	
		Grades 3–4	0.385 (0.018–3.431)	0.432	0.704 (0.327–1.475)	0.358
	Regional Lymph Node Involvement	No	Reference		Reference	
		Yes	0.231 (0.016–5.819)	0.284	0.620 (0.119–2.892)	0.539
	Distant Metastasis	No	Reference		Reference	
		Yes	0.12 (0.01–1.285)	0.07	0.165 (0.069–0.363)	<0.001
Multivariate Logistic Regression	Age Category	0–10	-		Reference	
		11–15			0.693 (0.213–2.094)	0.525
		16–21			0.565 (0.182–1.621)	0.302
	Sex	Male	-		Reference	
		Female			0.827 (0.412–1.667)	0.592
	Hispanic Ethnicity	No	Reference		Reference	
		Yes	NA (0–Inf)	0.996	0.342 (0.115–0.941)	0.043
	Insurance Status	Private insurance	-		Reference	
		Government			0.851 (0.413–1.771)	0.664
		Not insured			4.402 (0.525–98.341)	0.227
	Percentage of non-High School Graduates by Zip Code	≤10.8%	-		Reference	
		>10.8%			0.525 (0.202–1.346)	0.18
	Median Household Income by Zip Code	>$50,333	-		Reference	
		≤$50,333			1.389 (0.553–3.608)	0.489
	Maximum Tumor Dimension	≤8 cm	Reference		Reference	
		>8 cm	NA (0–Inf)	0.996	0.606 (0.279–1.281)	0.196
	Distant Metastasis	No	Reference		Reference	
		Yes	0.336 (0.008–18.286)	0.541	0.173 (0.068–0.407)	<0.001

## Data Availability

Access to this data is restricted. The data was sourced from the NCDB, a prospectively maintained repository developed through a collaboration between the Commission on Cancer (CoC) of the American College of Surgeons and the American Cancer Society. (Statement for NCDB Use: The data used in this study were sourced from the Commission on Cancer (CoC) of the American College of Surgeons and the American Cancer Society; however, these institutions have not verified, nor are they responsible for, the statistical validity of the data analysis or the conclusions drawn by the authors).

## Supplementary Materials

The following supporting information can be downloaded at: https://www.mdpi.com/article/10.3390/children11081025/s1, Supplementary Figure S1: Univariate Cox proportional hazards model evaluating the impact of all variables on the mortality risk for patients with vertebral osteosarcomas; Supplementary Figure S2: Univariate Cox proportional hazards model that assesses the impact of all variables on the mortality risk for patients with sacropelvic osteosarcomas; Supplementary Table S1: Factors associated with the odds of unplanned readmissions for patients who underwent surgical resection (CI, confidence interval; NA, not available; Inf, infinity); Supplementary Table S2: Characteristics associated with odds of prolonged length of stay (>7 days for vertebral and >20 days for sacropelvic osteosarcomas) for patients who underwent surgical resection (CI, confidence interval; NA, not available; Inf, infinity); Supplementary Table S3: Characteristics associated with the likelihood of receiving radiotherapy (CI, confidence interval; NA, not available; Inf, infinity); Supplementary Table S4: Characteristics associated with the likelihood of receiving chemotherapy (CI, confidence interval; NA, not available; Inf, infinity).
