# Supplementary material for: Exploring Pediatric Vertebral, Sacral, and Pelvic Osteosarcomas through the NCDB: Demographics, Treatment Utilization, and Survival Outcomes
Source: Children (Basel). 2024 Aug 21;11(8):1025. doi: 10.3390/children11081025 (PMC11353215; doi:10.3390/children11081025)
Supplement: Supplementary file 1 [file children-11-01025-s001.zip › Supplementary Figure 1.pdf]

Supplementary Figure S1. Univariate Cox proportional hazards model that assesses the impact of all variables on the mortality risk of patients with vertebral osteosarcomas.

		R	R
**Age Category**			*Reference*
**Sex**			*Reference*
**Race**			*Reference*
**Hispanic Ethnicity**			*Reference*
**Insurance Status**			*Reference*
**Percentage of non-High School Graduates in Zip Code**	≤		*Reference*
**Median Household Income of Zip Code**			*Reference*
	≤		
**Residing Population Size**	≥		*Reference*
**Charlson-Deyo Score**			*Reference*
	≥ 1		
**Maximum Tumor Dimension**	≤		*Reference*
**Regional Lymph Node Involvement**			*Reference*
**Distant Metastasis**			*Reference*
**Grade**			*Reference*
**Surgery - Residual Tumor**			*Reference*
**Radiotherapy**			*Reference*
**Chemotherapy**			*Reference*
