# Supplementary material for: Exploring Pediatric Vertebral, Sacral, and Pelvic Osteosarcomas through the NCDB: Demographics, Treatment Utilization, and Survival Outcomes
Source: Children (Basel). 2024 Aug 21;11(8):1025. doi: 10.3390/children11081025 (PMC11353215; doi:10.3390/children11081025)

Supplementary Figure S2. Univariate Cox proportional hazards model that assesses the impact of all variables on the mortality risk of patients with sacropelvic osteosarcomas.

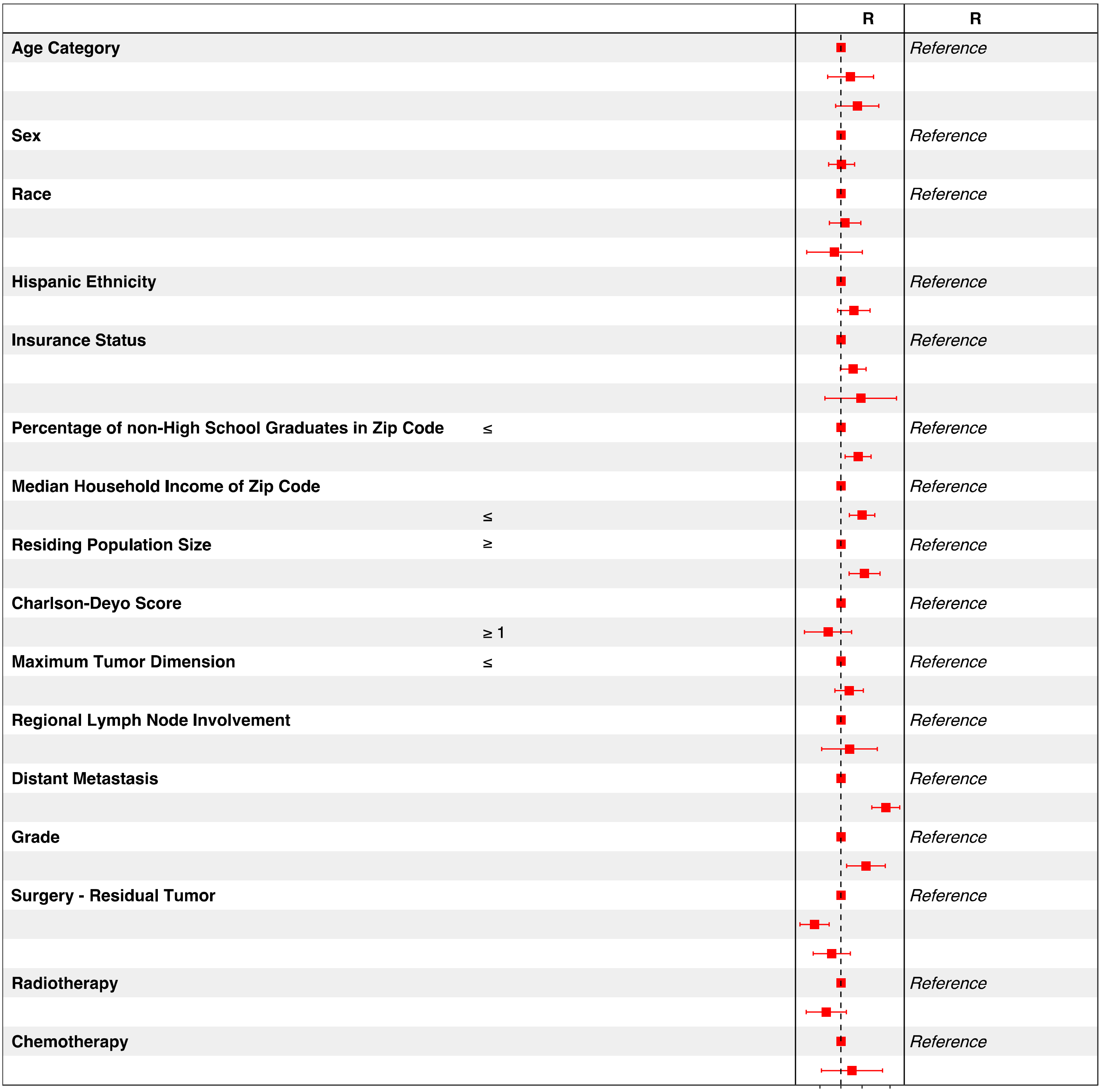

Supplement: Supplementary file 1 [file children-11-01025-s001.zip › Supplementary Figure 2.pdf]
