# Supplementary material for: Exploring Pediatric Vertebral, Sacral, and Pelvic Osteosarcomas through the NCDB: Demographics, Treatment Utilization, and Survival Outcomes
Source: Children (Basel). 2024 Aug 21;11(8):1025. doi: 10.3390/children11081025 (PMC11353215; doi:10.3390/children11081025)
Supplement: Supplementary file 1 [file children-11-01025-s001.zip › Supplementary Table 1.pdf]

Supplementary Table S1. Characteristics associated with odds of unplanned readmissions for patients who underwent surgical resection (CI, confidence interval; NA, not available; Inf, infinity).

	Variable		Vertebral		Sacropelvic	
			**Odds Ratio (95% CI)**	**p Value**	**Odds Ratio (95% CI)**	**p Value**
**Univariate Logistic Regression**	**Age Category**	0-10	*Reference*		*Reference*	
		11-15	0.364 (0.012 - 10.677)	0.508	NA (0 - Inf)	0.996
		16-21	0.889 (0.064 - 22.464)	0.931	NA (0 - Inf)	0.995
	**Sex**	Male	*Reference*		*Reference*	
		Female	0.167 (0.008 - 1.536)	0.146	0.545 (0.026 - 4.461)	0.606
	**Race**	White	*Reference*		*Reference*	
		Black	NA (0 - Inf)	0.996	4.063 (0.459 - 36.034)	0.177
		Other	3.167 (0.125 - 45.876)	0.401	NA (0 - Inf)	0.995
	**Hispanic Ethnicity**	No	*Reference*		*Reference*	
		Yes	NA (0 - Inf)	0.995	NA (0 - Inf)	0.994
	**Insurance Status**	Private insurance	*Reference*		*Reference*	
		Government	NA (0 - Inf)	0.996	1.862 (0.214 - 16.182)	0.545
		Not insured	-		NA (0 - Inf)	0.996
	**Percentage of non-High School Graduates in Zip Code**	≤ 10.8%	*Reference*		*Reference*	
		> 10.8%	0.467 (0.021 - 4.276)	0.534	0.357 (0.017 - 2.912)	0.381
	**Median Household Income of Zip Code**	> $50,333	*Reference*		*Reference*	
		≤ $50,333	NA (0 - Inf)	0.995	0.472 (0.023 - 3.856)	0.523
	**Population**	≥ 250,000	*Reference*		*Reference*	
		< 250,000	0.556 (0.025 - 5.128)	0.633	2.083 (0.099 - 17.868)	0.539
	**Charlson-Deyo Score**	0	*Reference*		*Reference*	
		≥ 1	NA (0 - Inf)	0.995	NA (0 - Inf)	0.994
	**Maximum Tumor Dimension**	≤ 8cm	*Reference*		*Reference*	
		> 8cm	2.429 (0.251 - 23.817)	0.418	NA (0 - Inf)	0.996
	**Grade**	Grade 1-2	*Reference*		*Reference*	
		Grade 3-4	0.846 (0.089 - 8.024)	0.877	0.208 (0.023 - 1.846)	0.131
	**Regional Lymph Node Involvement**	No	*Reference*		*Reference*	
		Yes	7.667 (0.259 - 234.428)	0.186	14.167 (0.569 - 199.748)	0.051
	**Distant Metastasis**	No	*Reference*		*Reference*	
		Yes	3.667 (0.145 - 52.872)	0.343	3.292 (0.153 - 29.448)	0.326
	**Residual tumor**	No residual tumor	*Reference*		*Reference*	
		Residual tumor	3.545 (0.390 - 77.561)	0.302	1.117 (0.054 - 9.278)	0.926
**Multivariate Logistic Regression**	**Sex**	Male	*Reference*		-	
		Female	0.158 (0.006 - 1.613)	0.155		
	**Race**	White	-		*Reference*	
		Black			2.720 (0.226 - 29.119)	0.389
		Other			NA (0 - Inf)	0.995
	**Grade**	Grade 1-2	-		*Reference*	
		Grade 3-4			0.303 (0.028 - 3.639)	0.304
	**Regional Lymph Node Involvement**	No	*Reference*		*Reference*	
		Yes	8.494 (0.234 - 413.551)	0.213	10.781 (0.337 - 213.576)	0.117
