# Supplementary material for: Exploring Pediatric Vertebral, Sacral, and Pelvic Osteosarcomas through the NCDB: Demographics, Treatment Utilization, and Survival Outcomes
Source: Children (Basel). 2024 Aug 21;11(8):1025. doi: 10.3390/children11081025 (PMC11353215; doi:10.3390/children11081025)
Supplement: Supplementary file 1 [file children-11-01025-s001.zip › Supplementary Table 2.pdf]

Supplementary Table S2. Characteristics associated with odds of prolonged length of stay (> 7 days for vertebral and > 20 days for sacropelvic osteosarcomas) for patients who underwent surgical resection (CI, confidence interval; NA, not available; Inf, infinity).

	Variable		Vertebral		Sacropelvic	
			**Odds Ratio (95% CI)**	**p Value**	**Odds Ratio (95% CI)**	**p Value**
**Univariate Logistic Regression**	**Age Category**	0-10	*Reference*		*Reference*	
		11-15	2.286 (0.224 - 53.429)	0.519	1.228 (0.272 - 6.658)	0.796
		16-21	0.889 (0.064 - 22.464)	0.931	0.920 (0.215 - 4.812)	0.913
	**Sex**	Male	*Reference*		*Reference*	
		Female	2.045 (0.345 - 16.749)	0.451	1.041 (0.339 - 3.053)	0.942
	**Race**	White	*Reference*		*Reference*	
		Black	4.500 (0.156 - 132.054)	0.322	0.219 (0.012 - 1.253)	0.16
		Other	9.000 (0.700 - 225.129)	0.102	0.656 (0.032 - 4.867)	0.716
	**Hispanic Ethnicity**	No	*Reference*		*Reference*	
		Yes	NA (0 - Inf)	0.994	1.083 (0.149 - 5.257)	0.926
	**Insurance Status**	Private insurance	*Reference*		*Reference*	
		Government	1.750 (0.275 - 10.597)	0.537	1.110 (0.335 - 3.451)	0.859
		Not insured	-		7.400 (0.646 - 169.168)	0.117
	**Percentage of non-High School Graduates in Zip Code**	≤ 10.8%	*Reference*		*Reference*	
		> 10.8%	2.000 (0.350 - 12.613)	0.436	1.339 (0.463 - 3.974)	0.59
	**Median Household Income of Zip Code**	> $50,333	*Reference*		*Reference*	
		≤ $50,333	1.393 (0.222 - 8.213)	0.712	1.217 (0.409 - 3.548)	0.718
	**Population**	≥ 250,000	*Reference*		*Reference*	
		< 250,000	2.000 (0.350 - 12.613)	0.436	2.802 (0.729 - 10.293)	0.12
	**Charlson-Deyo Score**	0	*Reference*		*Reference*	
		≥ 1	NA (0 - Inf)	0.995	1.083 (0.149 - 5.257)	0.926
	**Maximum Tumor Dimension**	≤ 8cm	*Reference*		*Reference*	
		> 8cm	4.000 (0.669 - 27.202)	0.133	1.314 (0.443 - 4.230)	0.63
	**Grade**	Grade 1-2	*Reference*		*Reference*	
		Grade 3-4	3.056 (0.519 - 25.105)	0.24	4.911 (0.872 - 92.601)	0.139
	**Regional Lymph Node Involvement**	No	*Reference*		*Reference*	
		Yes	NA (0 - Inf)	0.995	1.647 (0.074 - 18.239)	0.691
	**Distant Metastasis**	No	*Reference*		*Reference*	
		Yes	NA (0 - Inf)	0.994	0.794 (0.039 - 5.834)	0.841
	**Residual Tumor**	No residual tumor	*Reference*		*Reference*	
		Residual tumor	0.917 (0.147 - 5.257)	0.922	3.465 (1.043 - 11.477)	0.04
**Multivariate Logistic [illegible]**	**Race**	White	*Reference*		*Reference*	
		Black	4.015 (0.119 - 141.030)	0.393	0.220 (0.010 - 1.676)	0.212
		Other	11.507 (0.765 - 344.915)	0.092	0.444 (0.017 - 4.562)	0.537
	**Insurance Status**	Private insurance	-		*Reference*	
		Government			1.111 (0.272 - 4.241)	0.878
		Not insured			16.129 (1.141 - 564.389)	0.054
	**Population**	≥ 250,000	-		*Reference*	

		< 250,000			2.305 (0.514 - 9.939)	0.26
	**Maximum Tumor Dimension**	≤ 8cm	*Reference*		-	
		> 8cm	4.700 (0.663 - 44.252)	0.132		
	**Grade**	Grade 1-2	-		*Reference*	
		Grade 3-4			4.284 (0.533 - 122.786)	0.254
	**Residual Tumor**	No residual tumor	-		*Reference*	
		Residual tumor			4.389 (1.157 - 17.755)	0.031
