# Supplementary material for: Exploring Pediatric Vertebral, Sacral, and Pelvic Osteosarcomas through the NCDB: Demographics, Treatment Utilization, and Survival Outcomes
Source: Children (Basel). 2024 Aug 21;11(8):1025. doi: 10.3390/children11081025 (PMC11353215; doi:10.3390/children11081025)
Supplement: Supplementary file 1 [file children-11-01025-s001.zip › Supplementary Table 3.pdf]

Supplementary Table S3. Characteristics associated with odds of receiving radiotherapy (CI, confidence interval; NA, not available; Inf, infinity).

	Variable		Vertebral		Sacropelvic	
			Odds Ratio (95% CI)	p Value	Odds Ratio (95% CI)	p Value
Univariate Logistic Regression	Age Category	0-10	*Reference*		*Reference*	
		11-15	2.5 (0.264 - 56.744)	0.465	1.633 (0.369 - 11.449)	0.557
		16-21	3.125 (0.351 - 69.495)	0.356	2.105 (0.537 - 14.007)	0.347
	Sex	Male	*Reference*		*Reference*	
		Female	0.267 (0.05 - 1.255)	0.101	1.458 (0.627 - 3.394)	0.378
	Race	White	*Reference*		*Reference*	
		Black	6.333 (0.519 - 151.441)	0.159	0.847 (0.264 - 2.304)	0.759
		Other	6.333 (0.519 - 151.441)	0.159	1.084 (0.159 - 4.541)	0.921
	Hispanic Ethnicity	No	*Reference*		*Reference*	
		Yes	NA (0 - Inf)	0.994	0.658 (0.148 - 2.088)	0.521
	Insurance Status	Private insurance	*Reference*		*Reference*	
		Government	1.667 (0.328 - 8.229)	0.526	0.938 (0.386 - 2.188)	0.883
		Not insured	-		NA (0 - Inf)	0.989
	Percentage of non-High School Graduates in Zip Code	≤ 10.8%	*Reference*		*Reference*	
		> 10.8%	1.625 (0.35 - 7.699)	0.531	0.552 (0.228 - 1.281)	0.173
	Median Household Income of Zip Code	> $50,333	*Reference*		*Reference*	
		≤ $50,333	0.39 (0.069 - 1.827)	0.248	0.48 (0.178 - 1.165)	0.12
	Population	≥ 250,000	*Reference*		*Reference*	
		< 250,000	1.333 (0.267 - 6.37)	0.717	1.406 (0.433 - 3.908)	0.536
	Charlson-Deyo Score	0	*Reference*		*Reference*	
		≥ 1	NA (0 - Inf)	0.995	0.13 (0.05 - 0.282)	0.611
	Maximum Tumor Dimension	≤ 8cm	*Reference*		*Reference*	
		> 8cm	1.083 (0.219 - 5.063)	0.919	1.549 (0.613 - 4.466)	0.38
	Grade	Grade 1-2	*Reference*		*Reference*	
		Grade 3-4	1.364 (0.299 - 6.709)	0.691	1.05 (0.389 - 3.352)	0.928
	Regional Lymph Node Involvement	No	*Reference*		*Reference*	
		Yes	1.056 (0.046 - 12.5)	0.967	3.652 (0.711 - 15.94)	0.09
	Distant Metastasis	No	*Reference*		*Reference*	
		Yes	1.5 (0.172 - 10.873)	0.687	1.412 (0.512 - 3.55)	0.479
Multivariate Logistic Regression	Race	White	*Reference*		-	
		Black	6.333 (0.519 - 151.441)	0.159		
		Other	6.333 (0.519 - 151.441)	0.159		
	Percentage of non-High School Graduates in Zip Code	≤ 10.8%	-		*Reference*	
		> 10.8%			0.619 (0.18 - 1.934)	0.426
	Median Household Income of Zip Code	> $50,333	-		*Reference*	
		≤ $50,333			0.697 (0.197 - 2.439)	0.571
	Regional Lymph Node Involvement	No	-		*Reference*	
		Yes			4.050 (0.733 - 19.723)	0.085
