# Supplementary material for: Exploring Pediatric Vertebral, Sacral, and Pelvic Osteosarcomas through the NCDB: Demographics, Treatment Utilization, and Survival Outcomes
Source: Children (Basel). 2024 Aug 21;11(8):1025. doi: 10.3390/children11081025 (PMC11353215; doi:10.3390/children11081025)
Supplement: Supplementary file 1 [file children-11-01025-s001.zip › Supplementary Table 4.pdf]

Supplementary Table S4. Characteristics associated with odds of receiving chemotherapy (CI, confidence interval; NA, not available; Inf, infinity).

	Variable		Vertebral		Sacropelvic	
			Odds Ratio (95% CI)	p Value	Odds Ratio (95% CI)	p Value
Univariate Logistic Regression	**Age Category**	0-10	*Reference*		*Reference*	
		11-15	0.45 (0.02 - 4.221)	0.523	1.867 (0.233 - 12.066)	0.511
		16-21	1.1 (0.045 - 14.418)	0.943	1.214 (0.172 - 5.491)	0.817
	**Sex**	Male	*Reference*		*Reference*	
		Female	0.212 (0.01 - 1.503)	0.179	1.024 (0.313 - 3.587)	0.969
	**Race**	White	*Reference*		*Reference*	
		Black	NA (0 - Inf)	0.996	NA (0 - Inf)	0.992
		Other	NA (0 - Inf)	0.996	1.061 (0.179 - 20.306)	0.956
	**Hispanic Ethnicity**	No	*Reference*		*Reference*	
		Yes	NA (0 - Inf)	0.994	0.317 (0.091 - 1.266)	0.079
	**Insurance Status**	Private insurance	*Reference*		*Reference*	
		Government	1.406 (0.244 - 11.24)	0.715	0.923 (0.282 - 3.242)	0.896
		Not insured	-		NA (0 - Inf)	0.993
	**Percentage of non-High School Graduates in Zip Code**	≤ 10.8%	*Reference*		*Reference*	
		> 10.8%	1.964 (0.347 - 15.595)	0.467	0.203 (0.031 - 0.799)	0.043
	**Median Household Income of Zip Code**	> $50,333	*Reference*		*Reference*	
		≤ $50,333	0.75 (0.134 - 4.520)	0.741	0.536 (0.153 - 1.749)	0.304
	**Population**	≥ 250,000	*Reference*		*Reference*	
		< 250,000	1.176 (0.201 - 9.466)	0.863	NA (0 - Inf)	0.99
	**Charlson-Deyo Score**	0	*Reference*		*Reference*	
		≥ 1	NA (0 - Inf)	0.995	0.552 (0.13 - 3.793)	0.467
	**Maximum Tumor Dimension**	≤ 8cm	*Reference*		*Reference*	
		> 8cm	4 (0.561 - 81.649)	0.23	3.723 (1.131 - 13.163)	0.032
	**Grade**	Grade 1-2	*Reference*		*Reference*	
		Grade 3-4	4.444 (0.783 - 35.777)	0.111	2.276 (0.576 - 7.754)	0.203
	**Regional Lymph Node Involvement**	No	*Reference*		*Reference*	
		Yes	NA (0 - Inf)	0.994	NA (0 - Inf)	0.991
	**Distant Metastasis**	No	*Reference*		*Reference*	
		Yes	NA (0 - Inf)	0.995	NA (0 - Inf)	0.992
Multivariate Logistic Regression	**Sex**	Male	*Reference*		-	
		Female	0.212 (0.01 - 1.503)	0.179		
	**Hispanic Ethnicity**	No	-		*Reference*	
		Yes			0.493 (0.128 - 2.125)	0.312
	**Percentage of non-High School Graduates in Zip Code**	≤ 10.8%	-		*Reference*	
		> 10.8%			0.224 (0.032 - 0.975)	0.071
	**Maximum Tumor Dimension**	≤ 8cm	-		*Reference*	
		> 8cm			4.037 (1.189 - 14.754)	0.026
